# Supplementary material for: Effect of hydrogen on the integrity of aluminium–oxide interface at elevated temperatures
Source: Nat Commun. 2017 Feb 20;8:14564. doi: 10.1038/ncomms14564 (PMC5321721; doi:10.1038/ncomms14564)
Supplement: Supplementary Information — Supplementary Figures, Supplementary Notes and Supplementary References [file ncomms14564-s1.pdf]

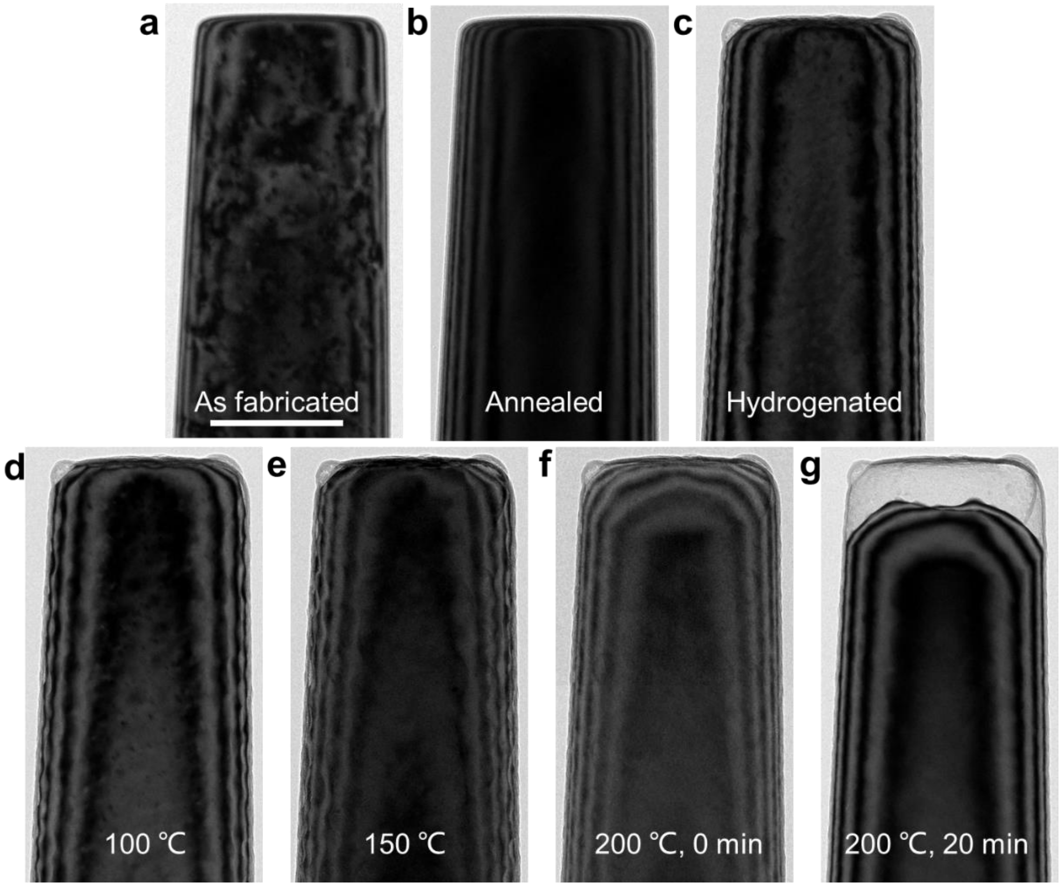

Supplementary Figure 1| Giant cavity formation process. (**a**) The as fabricated pillar contains lots of defects that are inherited from bulk sample and induced by FIB fabrication. (**b**) The pillar was thermal annealed at 200 °C in vacuum to remove the defects. (**c**) After hydrogenation, besides cavities formation, lots of small black spots (interstitial dislocation loops) formed inside the pillar. (**d**) At 100 °C, the proto-cavities grew up by coalescence with neighboring proto-cavities, while the dislocation loops remain unchanged. (**e**) At 150 °C, the size of the proto-cavities got slightly reduced, while the dislocation loops disappeared. (**f**) At 200 °C, the giant cavity grew up at the pillar top, all proto-cavities got refilled. (**g**) Keep at 200 °C for 20 min, the giant cavity continued to grew up even though all the proto-cavities are already refilled. Scale bar, 100 nm.

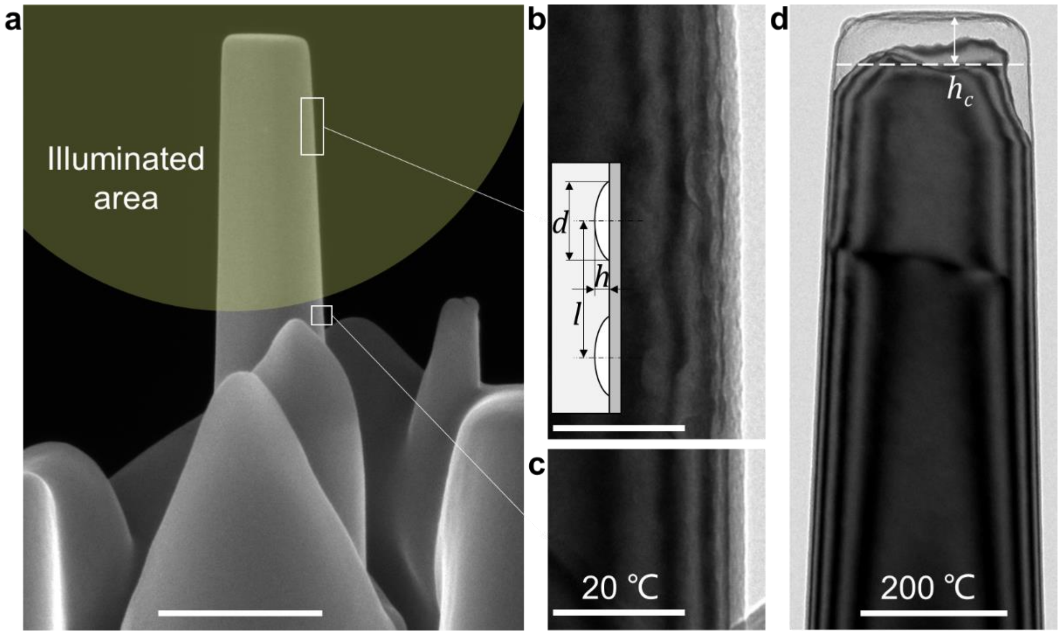

Supplementary Figure 2| The volume of the giant cavity. (**a**) The experimental setup showing that only the extruding part of the sample was hydrogenated (shown as the colored area). (**b**) TEM image of the hydrogenated area of the pillar. (**c**) TEM image of the un-hydrogenated area of the same pillar.(**d**) TEM image of the same pillar after heating. Scale bars, **a** 500nm, **b**& **c** 50 nm, **d** 200 nm, respectively.

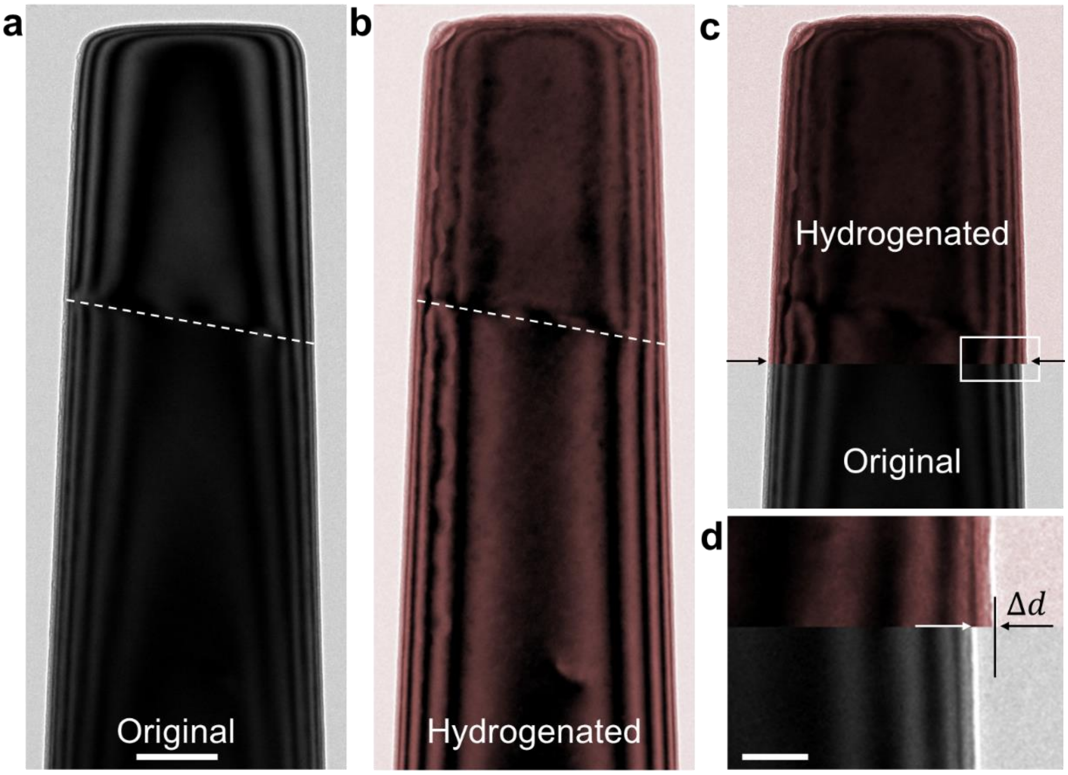

Supplementary Figure 3| Volume expansion during hydrogenation process. (**a**-**b**) TEM image of the pillar before and after hydrogenation, respectively. (**c**) Comparison of the diameter change at the position marked by the arrows before and after (colored area) hydrogenation, the images are aligned by the dislocation line marked by dashed lines in a and b. (**d**) Enlarged view of the boxed area in **c**, which shows the diameter increased by $\Delta d \approx 6.8$nm. Scale bars, **a** 100nm, **d** 20nm.

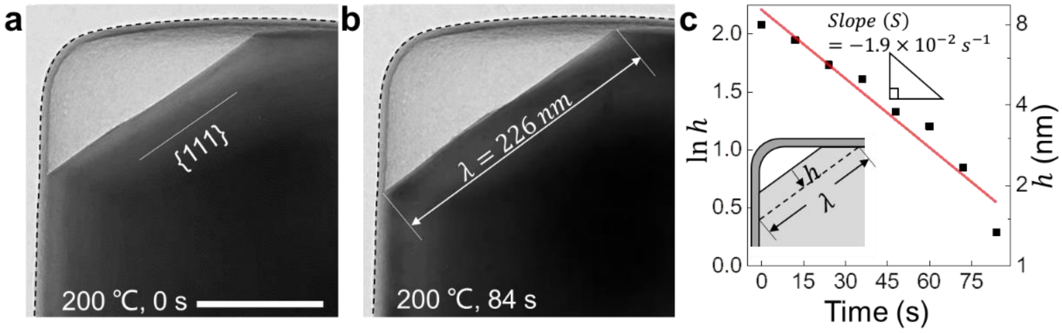

Supplementary Figure 4| Calculation of the diffusivity. (**a**) Upon reaching 200 °C, a small facet in the {111} plane was formed along the metal-oxide interface at the edge of the pillar. (**b**) The interface after being at 200 °C for 84 s shows the metal retreating with the {111}-faceted surface. Note the oxide maintains its shape throughout this process, as indicated by the dashed line. (**c**) The measured retreating distance h of the {111}-faceted surface versus time. Scale bar, 100 nm.

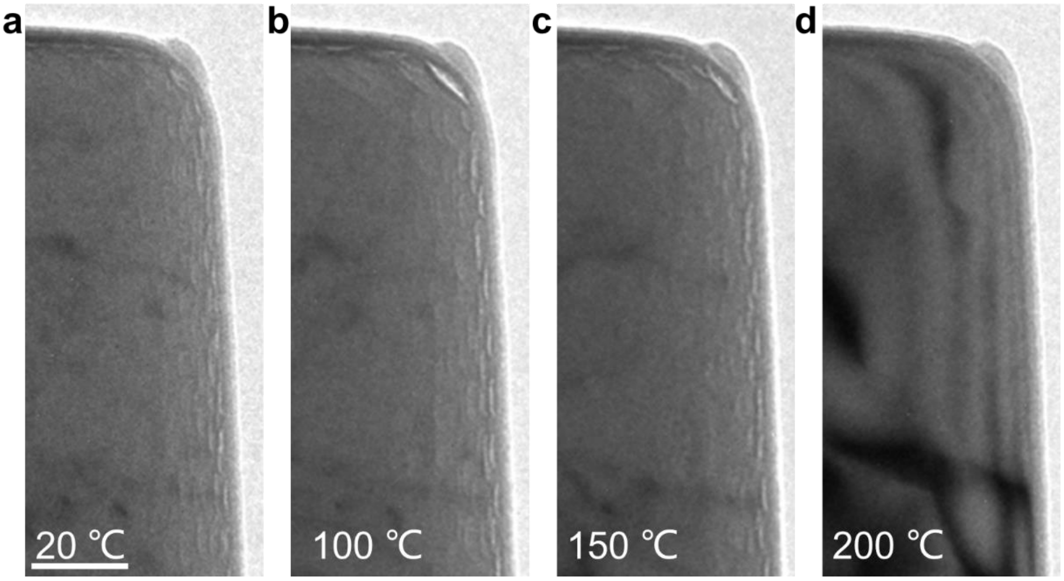

Supplementary Figure 5| Self-healing of a vacuum-aged sample during heating. (**a- d**) are snapshots of the sample at 20 °C, 100 °C, 150 °C, and 200 °C, respectively. From 20 °C to 100 °C, similar to that of the normal hydrogenated samples, the proto-cavities coalesced to form larger proto-cavities. From 100 °C to 150 °C, the proto-cavities became slightly refilled, and from 150 °C to 200 °C, instead of forming a giant cavity, all cavities were gradually refilled. Scale bar, 50 nm.

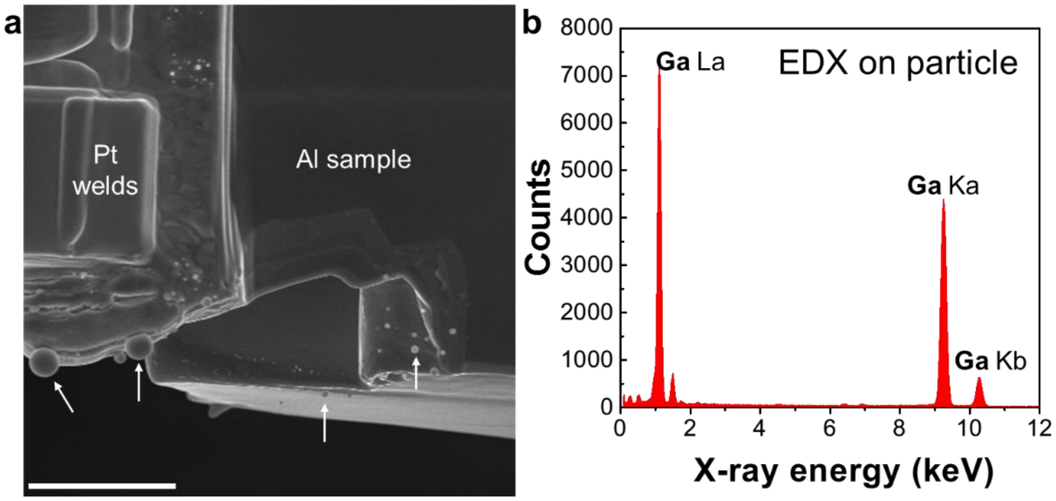

Supplementary Figure 6| Formation of Ga droplets after heating. (**a**) SEM image after thermal annealing. The implanted Ga segregated out and form tiny particles (marked by white arrows) on sample surface. (**b**) Energy dispersive X-ray analysis indicate that these particles are Ga. Scale bar, 5 μm.

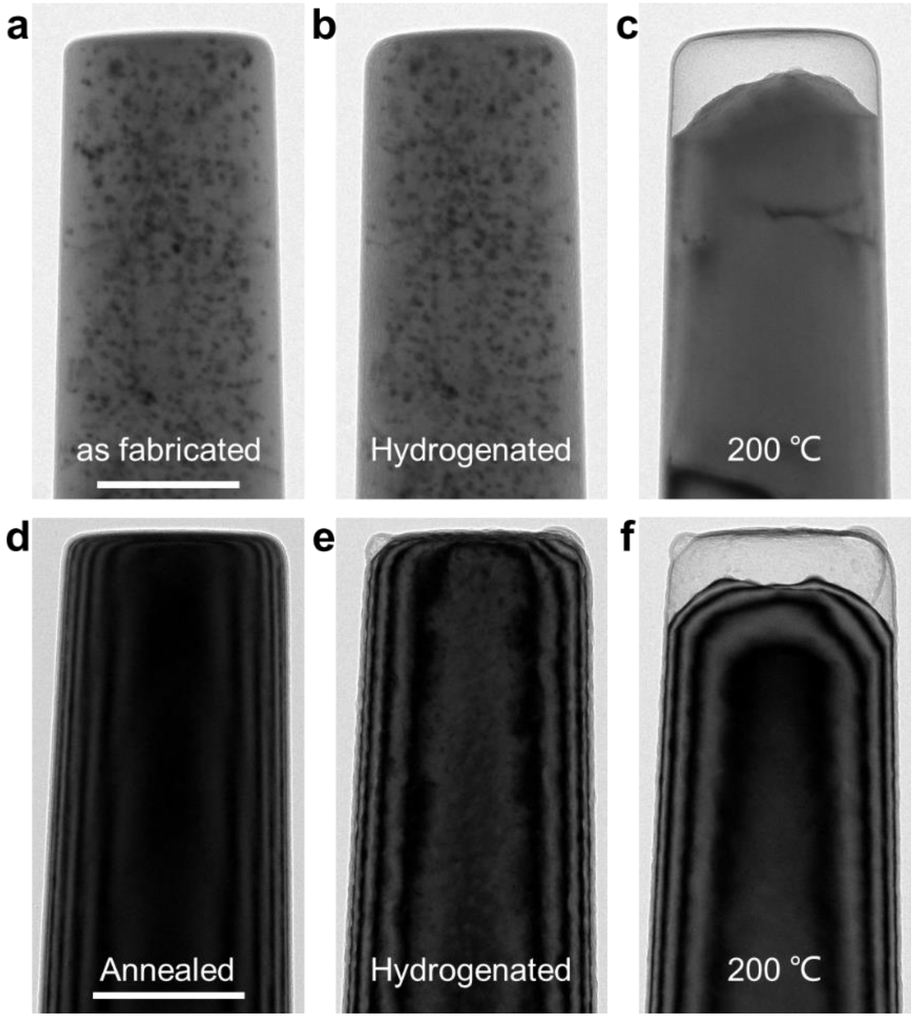

Supplementary Figure 7| The giant cavity formation on both as-fabricated and well-annealed aluminum samples. (**a**-**c**) giant cavity formation process in un-annealed sample. (**d**-**f**) giant cavity formation process in annealed sample, the FIB damage induced point defect clusters are removed by annealing before hydrogenation and heating. Scale bars, 200 nm.

Supplementary Note 1:

The MEMS heating chip. We designed and produced a MEMS heating chip specifically for samples prepared from bulk materials using FIB (Fig. 1). Unlike conventional MEMS heaters, which use a membrane-shaped heating area fixed in the center of the bulk handle[1,2], this heating chip has a free-standing thick heating block with a Si hotplate (Fig. 1 b). The hotplate is connected to the handle part of the chip with springs and has mounting bars to attach the sample at the free end. Serpentine metal filaments were deposited on the hotplate as the heater and the temperature sensor. Compared with traditional MEMS heaters, this design provides more convenience for transferring lift-out samples cut from bulk material. During heating, the springs can adaptively counterbalance the thermal expansion of the hotplate, preventing the bulging of the heating membrane that occurs in conventional MEMS heaters as well as minimizing sample drift. These springs can also be used to isolate thermal dissipation, helping to maintain an even temperature distribution in the hotplate with a low power supply. Finally, the small heating volume lends to a quick response with thermal equilibrium reached within a blink. The temperature is measured from the resistance of the metal coil using a calibrated temperature-resistivity relationship.

Supplementary Note 2:

Volume estimation. After normal hydrogenation, multiple giant cavities formed on both the pillar surface and the neighboring substrate area (see Fig. 4b), making reasonable

estimation of the cavity volume nearly impossible. To simplify this problem, we placed only the extruding part of the pillar into the irradiation zone (see Supplementary Fig. 2a). As can be seen from Supplementary Fig. 2b&c, after hydrogenation, small proto-cavities were evident only at the top and along the middle part of the pillar. The illuminated area can be seen as a cylinder with height (*H*=940 nm) and diameter (*D*=315 nm). The average size of the proto-cavities (*d*=30 nm, *h*=8.5 nm, *l*=50 nm, defined in the inset in Supplementary Fig.2b) after hydrogenation were measured from the TEM image. Assuming the proto-cavities are spherical in shape, the radius of the proto-cavities were calculated as $\mathrm{r} = \frac{(\frac{d}{2})^2+h^2}{2h} = 17.5\ nm$, and the average volume of each cavity was calculated as $\mathrm{V}_c = \pi \mathrm{h}^2 \left(r - \frac{h}{3}\right) = 3325\ nm^3$. SEM inspection shows that the proto-cavities distributed homogenously on the illuminated surface, hence the total volume of the proto-cavities could be estimated as $\mathrm{V}_{tot} = \mathrm{V}_c \left(\frac{\pi DH}{l^2}\right) = 1.2 \times 10^6\ nm^3$. After the same heating process up to 200 °C, only one giant cavity formed at the top of the pillar (Supplementary Fig. 2d); it had an estimated volume of $\mathrm{V}_{gc} = \pi \frac{D^2}{4} \mathrm{h}_c = 3.0 \times 10^6\ nm^3$. Therefore, the total volume of the proto-cavities was calculated at about 1/3 of the volume of the giant cavity.

If the addition volume was contributed by vacancies ($\mathrm{V}_v = \mathrm{V}_{gc} - \mathrm{V}_{tot} = 1.8 \times 10^6\ nm^3$), whereby the hydrogenated volume of $\mathrm{V}_{H2} = \pi \frac{D^2}{4} \mathrm{H} = 73.7 \times 10^6\ nm^3$ and the vacancy concentration was estimated as $\mathrm{C}_v = \frac{\mathrm{V}_v}{\mathrm{V}_{H2}} = 2.4 \times 10^4\ appm.$

Further, if the total volume is conserved, the pillar should swell up during the hydrogenation process, and this volume expansion should meet the volume of the final giant cavity. Assume the original diameter of the pillar is $D_0 = 315\ nm$, then the

original volume of the pillar is $V_0 = \pi H(\frac{D_0}{2})^2 = 7.27 \times 10^7\ nm^3$. After hydrogenation, the volume of the pillar should be expanded to $V_H = V_{gc} + V_0 = 7.38 \times 10^7\ nm^3$. Hence the diameter after hydrogenation should be $D_H = \sqrt{\frac{4V_H}{\pi H}} = 320.6\ nm$. As shown in Supplementary Fig. 3, the diameter at the marked point increased from 314.0 nm up to 320.8 nm, which meets good with the expectation.

Supplementary Note 3:

Diffusivity estimation. We used the same calculation methods used in our previous work [3]. To simplify the calculation, we only choose the period when the {111} facet was clear and nearly straight, retreating under a constant temperature of 200 °C. Supplementary Fig. 4a-b shows the starting and ending snapshots of the giant cavity in this calculation. Supplementary Fig. 4c plots the natural logarithm of the retreat height, which is a quasi-linear function of time (shown by the red line), giving a slope of *S*. Using the equation in Supplementary reference 3,the calculated diffusivity D_s for the giant cavity is $7.9 \times 10^{-8}\ cm^2\ s^{-1}$, close to the predicted value $3.5 \times 10^{-8}\ cm^2\ s^{-1}$ from the empirical rule at the same temperature[4].

Supplementary Note 4:

FIB effect on the experiment result. In aluminum, the damages caused by FIB fabrication are point defect clusters formed near sample surface and Ga implantation.[5,6] The point defect clusters are characterized to be interstitial Frank loops induced by

irradiation[6]. Both these FIB-induced defects and implanted Ga can be cleaned out by thermal annealing[7]. Supplementary Fig. 1a-b shows the results from thermal annealing at 200 °C. It can be seen that after annealing, the initially 'dirty' pillar became clean, as evidenced by the smooth thickness contour. Besides, we observed from SEM image that some spherical particles formed on the lamella surface after annealing, which was proved to be Ga by energy dispersive X-ray analysis (Supplementary Fig. 6).

To verify the FIB effect on the giant cavity formation process, annealed samples were hydrogenated and heated up in vacuum with the same experiment conditions mentioned in the method section. The comparison of results from as-fabricated pillar and well-annealed pillar are shown in Supplementary Fig. 7. After heating, giant cavities are formed in both samples, and the volumes of the giant cavities are comparable. This result indicates that the FIB-induced defects and Ga implantation have negligible effect on our observed cavity evolution.

Supplementary References:

1. van Huis, M. A. et al. Atomic imaging of phase transitions and morphology transformations in nanocrystals. Adv. Mater. 21, 4992–4995 (2009).
2. Allard, L. F. et al. A new MEMS-based system for ultra-high-resolution imaging at elevated temperatures. Microsc Res Tech 72, 208–215 (2009).
3. Xie, D.-G. et al. In situ study of the initiation of hydrogen bubbles at the aluminium metal/oxide interface. Nat. Mater. 14, 1–6 (2015).

4. Tan, C. M. & Roy, A. Electromigration in ULSI interconnects. Mater. Sci. Eng. R Reports 58, 1–75 (2007).

5. Mayer, J., Giannuzzi, L. a, Kamino, T. & Michael, J. TEM Sample Preparation and Damage. MRS Bull. 32, 400–407 (2007).

6. Idrissi, H. et al. Point Defect Clusters and Dislocations in FIB Irradiated Nanocrystalline Aluminum Films: An Electron Tomography and Aberration-Corrected High-Resolution ADF-STEM Study. Microsc. Microanal. 17, 983–990 (2011).

7. Kiener, D. et al. Advanced nanomechanics in the TEM: effects of thermal annealing on FIB prepared Cu samples. Philos. Mag. 92, 3269–3289 (2012)
